# The Importance of Antioxidant Micronutrients in Pregnancy

**DOI:** 10.1155/2011/841749

**Published:** 2011-09-13

**Authors:** Hiten D. Mistry, Paula J. Williams

**Affiliations:** ^1^Division of Women's Health, Maternal and Fetal Research Unit, King's College London, St. Thomas' Hospital, London SE1 7EH, UK; ^2^Human Genetics, School of Molecular and Medical Sciences, University of Nottingham, Queen's Medical Centre, Nottingham NG7 2UH, UK

## Abstract

Pregnancy places increased demands on the mother to provide adequate nutrition to the growing conceptus. A number of micronutrients function as essential cofactors for or themselves acting as antioxidants. Oxidative stress is generated during normal placental development; however, when supply of antioxidant micronutrients is limited, exaggerated oxidative stress within both the placenta and maternal circulation occurs, resulting in adverse pregnancy outcomes. The present paper summarises the current understanding of selected micronutrient antioxidants selenium, copper, zinc, manganese, and vitamins C and E in pregnancy. To summarise antioxidant activity of selenium is via its incorporation into the glutathione peroxidase enzymes, levels of which have been shown to be reduced in miscarriage and preeclampsia. Copper, zinc, and manganese are all essential cofactors for superoxide dismutases, which has reduced activity in pathological pregnancy. Larger intervention trials are required to reinforce or refute a beneficial role of micronutrient supplementation in disorders of pregnancies.

## 1. Introduction

### 1.1. Nutrition in Pregnancy

The importance of proper nutrition prior to and throughout pregnancy has long been known for optimising the health and well-being of both mother and baby [1]. Pregnancy is a period of increased metabolic demands with changes in a women's physiology and the requirements of a growing fetus [2, 3]. Insufficient supplies of essential vitamins and micronutrients can lead to a state of biological competition between the mother and conceptus, which can be detrimental to the health status of both [4] Deficiency of trace elements during pregnancy is closely related to mortality and morbidity in the new born [5]. Deficiencies of specific antioxidant activities associated with the micronutrients selenium, copper, zinc, and manganese can result in poor pregnancy outcomes, including fetal growth restriction [6], preeclampsia [7] and the associated increased risk of diseases in adulthood, including cardiovascular disease and type 2 diabetes [8–11]. 

A large body of research has investigated the role of macronutrients in pregnancy and especially the effects of both maternal under and over nutrition on the long-term health of the offspring [12, 13]. A number of hypotheses have been suggested to explain the contribution of maternal nutrition during the fetal and embryonic periods and the programming of the cardiovascular and metabolic system of the offspring [14, 15]. In addition to the contribution of macronutrition to successful pregnancy more recently studies have begun to focus on the role of essential specific micronutrients, so called because they are an absolute requirement and are required in only small amounts daily [16]. 

The pregnant mother must provide a source of nourishment and gaseous exchange to enable maximal embryonic and fetal growth to occur, whilst at the same time preparing her body for labour and parturition and the later demands of lactation. In addition a careful balance must be maintained between providing immune surveillance to protect the mother from infection and whilst at the same time allowing the implantation and survival of the semiallogenic conceptus [17, 18]. The development and establishment of the placenta and its circulatory system is crucial in the successful maintenance of maternal health and also enabling development of the embryo and growth of the fetus.

**Figure 1:**
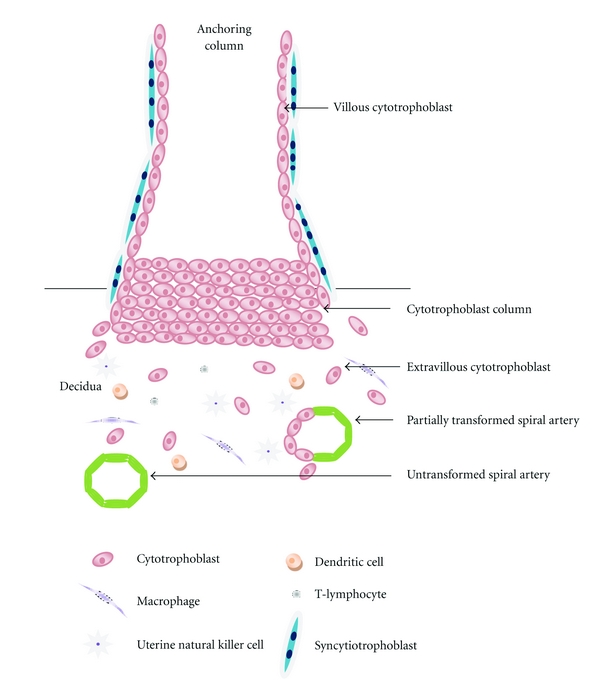
Diagram of the maternal fetal interface in early pregnancy. Extravillous cytotrophoblast cells migrate away from the cell column of the anchoring villus and invade through the maternal deciduas and inner third of the myometrium in order to gain access to maternal blood supply via maternal spiral arteries. Transformation of the maternal spiral arteries occurs as endovascular trophoblast help convert the endothelial cells lining the arteries into an amorphous fibrinoid matrix which is unresponsive to vasoactive stimuli and serves to enlarge the vascular lumen maximising blood supply to the intervillous space.

**Figure 2:**
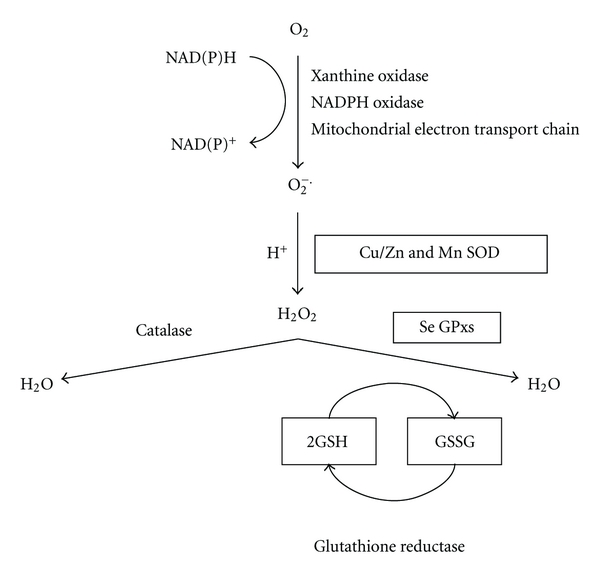
Major pathways of reactive oxygen species generation and metabolism. Superoxide can be generated by specialised enzymes, such as the xanthine or NADPH oxidases, or as a byproduct of cellular metabolism, particularly the mitochondrial electron transport chain. Superoxide dismutase (SOD), both Cu/Zn and Mn SOD, then converts the superoxide to hydrogen peroxide (H_2_O_2_) which has to be rapidly removed from the system. This is generally achieved by catalase or peroxidases, such as the selenium-dependent glutathione peroxidases (GPxs) which use reduced glutathione (GSH) as the electron donor.

The establishment of the fetoplacental circulation requires the invasion of placental derived extravillous trophoblast from anchoring cytotrophoblast columns through the maternal decidua and into the maternal spiral arteries (Figure 1). During the first 8 weeks of pregnancy, trophoblast plugs within the spiral arteries which exist to protect embryonic DNA from damage by oxidative stress [19] are released allowing the onset of placental circulation (Figure 1). The release of trophoblast plugs with flow of blood into the intervillous space leads to the generation of oxidative stress [20]. However, the placenta is armed with antioxidant defences, including the selenium-dependent glutathione peroxidases, thioredoxin reductases, selenoprotein-P, and copper/zinc and manganese superoxide dismutases (Cu/Zn and Mn SODs) (Figure 2), which protect the placenta from any undue harm [21–24]. As the extravillous trophoblast cells migrate through the spiral artery wall, they are involved in the process of physiological conversion, which serves to enlarge the vessel lumen thereby maximising blood supply to the intervillous space, enabling maximal perfusion of nutrients and gases through the placental syncytiotrophoblast. Deficient extravillous trophoblast invasion is associated with reduced spiral artery remodelling leading to reduced blood flow and increased oxidative stress within the placenta and is known to be the primary defect occurring in preeclampsia [25, 26], fetal growth restriction [27, 28], and sporadic miscarriage [29]. Additionally, preeclampsia has also been associated with reduced levels of antioxidant enzyme protection causing further placental damage [30–32].

The pathogenesis of adverse pregnancy outcomes including preeclampsia and fetal growth restriction [33] and a number of neonatal outcomes [34] has been shown to be associated with oxidative stress. Preeclampsia (*de novo* proteinuric hypertension) is estimated to occur in ~3% of all pregnancies and is a leading cause of maternal and perinatal mortality and morbidity in the Western world [35, 36]; together with other hypertensive disorders of pregnancy, preeclampsia is responsible for approximately 60,000 maternal deaths each year [37] and increases perinatal mortality five-fold [38]. Optimal outcome for the mother and child often dictates that the infant is delivered early leading to increased preterm delivery and low infant birth weight rates. Placental and maternal systemic oxidative stress are components of the syndrome [39] and contribute to a generalised maternal systemic inflammatory activation [40]. Placental ischaemia-reperfusion injury has been implicated in excessive production of ROS, causing release of placental factors that mediate the inflammatory responses [41]. 

Fetal growth restriction is associated with increased perinatal mortality and morbidity [42]. The mechanisms are still to be elucidated but a likely factor is placental ischemia/hypoxia [43], but it is thought that ischemia-reperfusion injury may contribute to the oxidative stress and could result in the release of reactive oxygen species into the maternal circulation possibly resulting in oxidative DNA damage and may underlie development of fetal growth restriction [44].

The current paper focuses specifically on those micronutrients which are associated with antioxidant activity due to the importance of oxidative stress in both normal pregnancy and pathological pregnancy outcomes. However, it is important to highlight that other micronutrients are also important in pregnancy [16] but are outside the scope of the current paper.

## 2. Methods

A number of micronutrients and vitamins are known to serve as antioxidants or be essential cofactors for antioxidant enzymes; these include selenium, copper, zinc, manganese, and vitamins C and E. The present paper summarises current understanding of the importance of these micronutrient antioxidants in pregnancy. For this paper, we included data and relevant information obtained through a search of the PubMed database using free text and Medical Subject Headings terms for all articles published in English from 1971 through 2011 which included the term “selenium,” “zinc,” “copper,” “manganese,” “vitamin E and C,” and one of the following: “pregnancy,” “preeclampsia,” or “fetal growth restriction.” We further included published and unpublished data from our own laboratories and an “in-house” library of relevant publications. The procedure was concluded by the perusal of the reference sections of all relevant studies or reviews, a manual search of key journals and abstracts from the major annual meetings in the field of pregnancy, and nutrition and contact with experts on the subject, in an effort to identify relevant unpublished data. 

### 2.1. Selenium

Selenium is incorporated into proteins to make selenoproteins, including the glutathione peroxidase antioxidant enzymes, thioredoxin reductases, and selenoprotein-P [45]. In addition, selenium is essential for the production of active thyroid hormones and is essential for normal thyroid function [46]. Maternal selenium concentrations and glutathione peroxidase activity fall during pregnancy (selenium concentrations in 1st trimester: 65 *μ*g/L; 3rd trimester: 50 *μ*g/L) [47, 48]. Worldwide differences exist in assessment of selenium requirements, adequacy, and intakes (Table 1). Most of these values have been determined from the intake believed necessary to maximise the activity of the antioxidant glutathione peroxidase in plasma [49, 50] and selenium, like vitamins C and E which have received much attention in recent years. It has been observed that babies on average have lower selenium concentrations compared to the mother (maternal selenium 58.4 *μ*g/L; umbilical cord selenium: 42.1 *μ*g/L) [32, 51], which is expected as selenium is transported via the placenta across a concentration gradient via an anion exchange pathway, shared with sulphate [52, 53]. 

Recurrent early pregnancy loss has been associated with reduced serum selenium concentrations compared to healthy controls in two observational studies from UK (mean ± SD: 54 ± 19 versus 76 ± 14 *μ*g/L, resp.) [54] and Turkey (55 ± 17 versus 81 ± 16 *μ*g/L, resp.) [55]. It has therefore been suggested that reduced selenium concentration results in reduced glutathione peroxidase activity culminating in reduced antioxidant protection of biological membranes and DNA during the early stages of embryonic development [54, 56]. Although speculative, and requiring larger placebo-controlled randomised trials, women with recurrent early pregnancy loss may benefit from optimisation of selenium status.

Recently, our group and others have demonstrated, through retrospective studies, the association between low serum selenium concentrations and reduced antioxidant function of the associated antioxidant glutathione peroxidase enzymes in women with preeclampsia (defined a *de novo* proteinuric hypertension) [32, 57, 58]. It has been suggested that adequate selenium status is important for antioxidant defence and may be a potential factor in women at risk of preeclampsia; this hypothesis has been further justified by the reduced expression and activities of glutathione peroxidase found in maternal, fetal, and placental samples taken from 25 preeclamptic pregnancies, when compared to 27 normal controls in our recent cross-sectional retrospective study [32]. Dawson et al. also completed a retrospective study in the USA and reported lower amniotic fluid selenium concentrations in 29 preeclamptics delivering between 33- and 36-week gestation compared to 48 gestation-matched controls (10 ± 1 versus 7 ± 0.7 *μ*g/L, resp.) [59].

Fetal growth restriction or delivery of a small for gestational age (SGA) is defined as an individualised birth weight ratio below the 10th percentile [42]. Reports of selenium concentrations with regards to fetal growth restriction are inconsistent. A retrospective study had reported low placental selenium concentrations in 49 mothers affected by fetal growth restriction, compared to 36 healthy normal birth weight controls [60], whereas others have reported higher [61, 62] or unchanged concentrations [63]. Another retrospective study also demonstrated in 81 SGA babies, infant plasma selenium concentrations to be significantly lower compared to controls [64]. A recent retrospective study by our group on an adolescent cohort [65] found lower maternal plasma selenium concentrations on 28 mothers who delivered SGA babies compared to 143 healthy controls [66]. Further studies are warranted to fully investigate the potential link between selenium deficiency and fetal growth restriction.

Only a small number of selenium supplementation trials during pregnancy have been carried out to date. A prospective, randomised, placebo-controlled study of selenium supplementation during and after pregnancy in pregnant women positive for thyroid peroxidase antibodies (TPOAb(+)) who are prone to develop postpartum thyroid dysfunction (PPTD) and permanent hypothyroidism found that supplementation with 200 *μ*g/d selenomethionine significantly reduced the incidence of PPTD and permanent hypothyroidism [67]. The authors concluded that selenium supplementation during pregnancy and in the postpartum period reduced thyroid inflammatory activity and the incidence of hypothyroidism, possibly by increasing the selenoproteins glutathione peroxidase or iodothyronine deiodinase activities, thereby contributing in part, to counterbalance the postpartum immunological rebound [67]. 

To date there have been a couple of small placebo-controlled randomised control trials on selenium supplementation, reporting lower rates of preeclampsia and/or pregnancy-induced hypertension in the supplemented groups [68–70]. It must be noted that neither of these studies adequately addressed the role of supplementation on the incidence of preeclampsia. Currently, the “Selenium in Pregnancy Intervention Trial” (SPRINT) is underway in the UK, jointly by the University of Surrey and Oxford. This is a small randomised controlled trial of selenium supplementation (60 *μ*g a day). It is not powered to demonstrate clinical benefit but will provide insight into the impact of selenium supplements on laboratory measurements of circulating factors that are relevant to the development of preeclampsia. If this is successful, a much larger multicentre trial will be needed to analyse clinical benefit.

### 2.2. Copper

Copper is an essential cofactor for a number of enzymes involved in metabolic reactions, angiogenesis, oxygen transport, and antioxidant protection, including catalase, superoxide dismutase (SOD) (Figure 2) and cytochrome oxidase [71]. During pregnancy, plasma copper concentrations significantly increase, returning to normal nonpregnant values after delivery (Mean ± SD–1st trimester: 147.6 ± 34.6; 3rd trimester: 204.2 ± 41.8 *μ*g/L) [72–74]. The increase in copper with progression of pregnancy could be partly related to synthesis of ceruloplasmin, a major copper-binding protein, due to altered levels of oestrogen [74]. Approximately 96% of plasma copper is strongly bound to ceruloplasmin [75], a protein with antioxidant ferroxidase properties [75, 76]. The dietary intake of copper in women aged between 19 and 24 years is generally below the recommended levels (Table 1) [77] which may cause problems during pregnancy when requirements increase [78].

Copper is essential for embryonic development [82]. Maternal dietary deficiency can result in both short-term consequences, including early embryonic death and gross structural abnormalities, and long-term consequences such as increased risk of cardiovascular disease and reduced fertilisation rates [71, 83]; current recommendations on intakes are summarised in Table 1. Severe copper deficiency can lead to reproductive failure and early embryonic death [84], whereas mild or moderate deficiency has little effect on either the number of live births or neonatal weight [85].

Cu/Zn SOD is an important antioxidant known to be expressed in both maternal and fetal tissues [86]. Copper concentration has been shown to be higher in maternal plasma than in umbilical cord plasma [87–89]. It has been suggested that the placenta acts as a blockade in the transfer of copper from the mother to the fetus [89, 90]. A recent observational Turkish study on 61 placentae from healthy pregnancies between 37- and 40-week gestation found that copper concentrations positively correlated with neonatal weight; the authors suggested that copper may have interactive connections in human placenta [91] and this requires further studies to fully elucidate its role. It is known that copper is transferred across the placenta via high-affinity copper transporter (CTR1) and has been shown to be expressed early in pregnancy, and it is also thought that placental copper transport is related to iron transport but the mechanism is unknown [78].

A small retrospective study in the 1980s reported increased placental copper concentrations in 8 preeclamptic women compared to 10 controls (53 versus 124 *μ*g/Kg, resp.), suggesting that this increase in preeclampsia maybe an exaggerated response of normal pregnancies [92]. Further retrospective studies from Turkey have shown elevation of maternal serum copper levels in preeclampsia after clinical onset of the disease compared to controls (mean ± SD: 159 ± 38 versus 194 ± 52 *μ*g/L, resp.) [93, 94]. Moreover, increased amniotic fluid copper concentration from 19 preeclamptic women compared to 53 controls has also been reported (mean ± SD: 19 ± 5 versus 29 ± 3 *μ*g/L, resp.) [59]. It is thought that as copper is a redox-active transition metal and can participate in single electron reactions and catalyse the formation of free radicals, including undesirable hydroxyl radicals, it could contribute to oxidative stress characteristic of preeclampsia [93]. This illustrates that copper itself appears to act as a pro-oxidant, but when associated in Cu/Zn SOD functions as an antioxidant. Furthermore, studies have reported increased levels of serum ceruloplasmin in women with preeclampsia; this positively correlated with serum malondialdehyde, suggesting an increased production of this antioxidant protein in response to increased lipid peroxidation [93, 95, 96], although further studies are required to confirm this hypothesis. However, it must be remembered that concentrations early in pregnancy have yet to be determined and data on copper status in normal human pregnancies are sparse. This is further hampered by the fact that at present there is no reliable biomarker for copper status, so whether deficiency is a significant public health problem remains unclear [97].

### 2.3. Zinc

Zinc is an essential constituent of over 200 metalloenzymes participating in carbohydrate and protein metabolism, nucleic acid synthesis, antioxidant functions (through Cu/Zn SOD; Figure 2), and other vital functions such as cellular division and differentiation, making it essential for successful embryogenesis. [72]. It was estimated in 2002 by the World Health Organisation that suboptimal zinc nutrition effected nearly half the world's population [98]. During pregnancy, zinc is also used to assist the fetus to develop the brain and also to be an aid to the mother in labour [99]. It has been estimated that the total amount of zinc retained during pregnancy is ~100 mg [100]. The requirement of zinc during the third trimester is approximately twice as high as that in nonpregnant women [81]. Plasma zinc concentrations decline as pregnancy progresses (mean ± SD–1st trimester: 71.3 ± 12.9 *μ*g/L; 3rd trimester: 58.5 ± 11.5 *μ*g/L) [72, 74, 101, 102]. In addition, a nutrition analysis revealed that in pregnant women the everyday diet intakes include not more than 50% of the daily requirement of zinc [103].

Alteration in zinc homeostasis may have devastating effects on pregnancy outcome, including prolonged labour, fetal growth restriction, or embryonic or fetal death [104]. Zinc is a trace element with a great importance for fetal growth restriction where it is used in order to improve fetal growth [72]. Goldenberg et al. completed a randomised double-blind placebo-controlled trail of zinc supplementation (25 mg per day) during pregnancy from 19-week gestation in African-American women (294 in supplemented and 286 in placebo group). Those given zinc supplementation had a significantly greater birth weight and head circumference compared to the placebo group highlighting the importance of adequate zinc supply during pregnancy [105]. A limitation of many of the randomised controlled trails of zinc supplementation and pregnancy outcome is that they lacked the sample sizes to detect differences [104].

Many of the zinc supplementation studies have been conducted in developing countries where incidence of zinc deficiency is high, and these women are often selected as they are less well nourished or have low plasma zinc levels [105, 106]; benefits of supplementation include reduced incidence of pregnancy-induced hypertension or low birth weights [105]. These studies do however suggest the benefits of zinc supplementation in developing countries where zinc deficiency is likely, although for developed countries there is conflicting data as to the benefits [107–110]. 

Zinc deficiency has been associated with preeclampsia since the 1980s [59, 92, 111] including adolescent pregnancies [112]. Placental zinc concentration has also been shown to be lower in preeclampsia in cross-sectional retrospective studies with placental zinc values positively correlating with birth weights [92, 113, 114]. More recently lower serum concentrations of zinc in preeclampsia compared to controls have been shown in two relatively small retrospective studies from Turkey (mean ± SD: 10.6 ± 4.4 versus 12.7 ± 4.1 *μ*g/L, resp.) [94, 115]; the authors suggested that this may be useful for early diagnosis as lower plasma zinc concentrations have been associated with increased lipid peroxidation in rat studies [116]. Moreover, in a recent retrospective study in India, reduced serum zinc concentrations in mild and severe preeclamptic mothers compared to controls were reported; the authors suggest that the reduction could not only effect the antioxidant protection but could also contribute to the rise in blood pressure [117]. The lower serum zinc concentrations in mothers who develop preeclampsia have been suggested to at least be partly due to reduced oestrogen and zinc binding-protein levels [118]. Zinc is transported across the placenta via active transport from the mother to the fetus [111]. Studies have shown that the fetus has notably higher zinc concentrations compared to the mother, even in cases of preeclampsia [111, 119], indicating that the fetus, itself, can maintain adequate zinc homeostasis. Amniotic fluid zinc concentrations have also been reported to be decreased in preeclamptic women delivering preterm (33- to 36-week gestation) in a small retrospective cross-sectional study from the USA [59]. It must be noted that as with all these micronutrients, the concentrations early in pregnancy in relation to the development of pregnancy complications remains to be established.

### 2.4. Manganese

Manganese is a free element in nature (often in combination with iron); furthermore, manganese(II) ions function as cofactors for a number of enzymes; the element is thus a required trace mineral for all known living organisms. Manganese is also an important cofactor for a number of enzymes, including the antioxidant manganese superoxide dismutase (Mn-SOD; Figure 2) which may protect the placenta from oxidative stress by detoxifying superoxide anions [120]. Dietary manganese is the main source of exposure under normal circumstances; current requirements for manganese, although less studied compared to other micronutrients, are shown in Table 1 and little is known about the effects of deficiency or excess of manganese on the developing human fetus or pregnancy outcome [121]. This is further hampered by the fact that at present sensitive biomarkers of manganese exposure and nutritional status are not available other than some estimates from blood concentrations [121]. 

Circulating whole blood manganese concentrations have been shown to be lower in women with fetal growth restriction compared to healthy controls (mean ± SD: 16.7 ± 4.8 versus 19.1 ± 5.9 *μ*g/L, resp.) indicating that this micronutrient may be important in maintaining fetal growth [122]. This study also found that manganese concentrations were higher in umbilical samples from fetal growth restriction cases compared to controls suggesting that manganese contributes to different effects on birth weight in healthy mothers [122]. Zota et al. retrospective study in the USA reported a nonlinear relationship between manganese concentrations and birth weights in a cohort of 470 full-term (delivered at >37-week gestation) infant further indicating the potential affect on fetal growth [123]. A recent small retrospective study of African-American mothers reported reduced umbilical cord whole blood manganese concentrations in neonates born to mothers with preeclampsia compared to controls (mean (95% CI): 2.2 (1.5, 3.2) versus 3.7 (3.2, 4.2) *μ*g/L, resp.) [124]. Furthermore, this study found that like other micronutrients umbilical cord blood from smoking mothers had reduced manganese concentrations [124]. Than et al. demonstrated increased fetal membrane MnSOD mRNA expression in women with preterm labour [125]. Manganese is one of the least studied micronutrients, and at present no supplementation trial has been published which may reflect the lack of data on manganese concentrations in pregnancy.

### 2.5. Vitamins C and E

Vitamin C (ascorbic acid and dehyroascorbic acid) is an essential water-soluble vitamin found widely in fruit and vegetables; it has important roles in collagen synthesis, wound healing, prevention of anaemia, and as an antioxidant as it can quench a variety of reactive oxygen species and reactive nitrogen species in aqueous environments [126]. Vitamin C is commonly included in low doses (<200 mg/day) within multivitamin preparations for pregnancy but has also been given in higher doses (up to 1000 mg/day) as a supplement, alone or in combination with vitamin E [127]. Smoking has been shown to increase oxidative stress and metabolic turnover of vitamin C, thus the requirement for smokers is increased by 35 mg/day [79]. 

**Figure 3:**
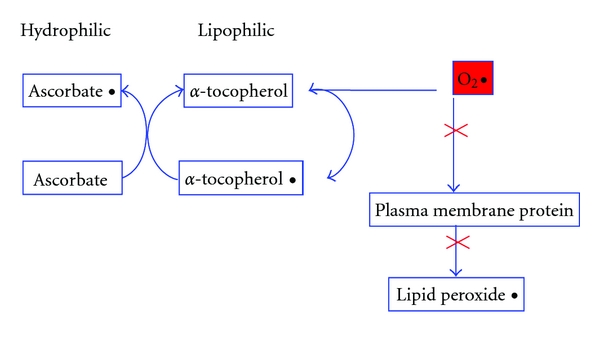
Synergistic mechanisms of vitamin C (ascorbate) and vitamin E (*α*-tocopherol) to prevent lipid peroxidation. O_2_ **^•^**: oxygen free radical.

Vitamin E (*α*-tocopherol) is a lipid-soluble vitamin acting with the lipid membrane and with synergistic interactions with vitamin C [128] (Figure 3). Vitamin E functions primarily as a chain-breaking antioxidant that prevents propagation of lipid peroxidation [127, 129]. A considerable interest exists regarding prevention of maternal and perinatal morbidity with vitamins C and E. However, the most recent meta-analysis of ten trials (6533 women) published in 2008 of antioxidant supplementation (including vitamin C and E but also other supplements such as lycopene) showed no difference in the relative risk (RR) of preeclampsia (RR 0.73, 95% CI 0.51 to 1.06), preterm birth (before 37 weeks) (RR 1.10, 95% CI 0.99 to 1.22), SGA infants (RR 0.83, 95% CI 0.62 to 1.11), or any baby death (RR 1.12, 95% CI 0.81 to 1.53) [7]. Considerable heterogeneity between the trials was seen reflecting the different supplements studied, the varying risk criteria used for entry into the studies, and the study sizes. A couple of subsequent recent multicentre double-blinded randomised trials of a combination of vitamin C and E [130, 131] also found that supplementation did not reduce the rate of preeclampsia or gestational hypertension and, like the Vitamins In Preeclampsia trial in 2006 [132], increased the risk of fetal loss or perinatal death and preterm prelabour rupture of membranes. Another recent multicentre placebo-controlled trial of vitamin C and E in women with type-1 diabetes in pregnancy (DAPIT) also reported no differences in the rates of preeclampsia between supplemented or placebo groups [133]. Further investigations are required as the concentrations of these vitamins remain significantly reduced in women with preeclampsia, but in the absence of further evidence, routine supplementation with higher dose vitamin C and E is not recommended as they can be potentially dangerous in high concentrations.

## 3. Adolescent Pregnancies

Another factor to highlight is that pregnancy in adolescent women is a topic of increasing health concern in many countries [134]. Pregnant adolescents are of a greater nutritional risk as they themselves are undergoing an intense process of growth and development [135]. It has been suggested that there is competition for nutrients between a pregnant adolescent and her fetus, as both are in critical stages of growth during the gestational period [65, 136]. As mentioned throughout above, micronutrient deficiencies have been associated with adolescent pregnancies [66, 87, 112]. The recent About Teenage Eating study showed that teenage pregnancy in the UK is associated with decreased consumption of micronutrients and that this is associated with an increased risk of SGA infants [65]. 

Low micronutrient intake poses a health risk to successful pregnancy in the UK, especially in adolescence. Socioeconomic deprivation in the UK population has been shown to be an independent risk factor for eating less fruit and vegetables and also for increased smoking, which is known to cause further depletion of micronutrients [137]. Although attempts are being made to persuade women of low socioeconomic status of the importance of a balanced diet, the central roles that fruit and vegetables have in this and that healthy food can be low cost and convenient, this is often met with resistance [138]. 

The increase in pregnancies in mothers of older ages highlights another subgroup at increased risk of pregnancy complications. We are not aware of any studies at present examining this important group, and future investigations are required especially as some of these micronutrient concentrations decline with increasing age.

## 4. Conclusions

Increased knowledge about the importance of these specific antioxidant micronutrients and the crucial part that they have in maintaining successful pregnancy and determining both the long- and short-term health of both mother and baby needs to be addressed and made a key focus for future health strategies in improving pregnancy outcomes. This is particularly important with regards to preeclampsia and fetal growth restriction, where oxidative stress is an essential component to the aetiology of these conditions and so these specific antioxidant micronutrient deficiencies may play a contributing role. Only by fully understanding the requirements for micronutrients during pregnancy will we be able to evaluate the potential use of these dietary antioxidant supplements as a way of preventing pathological pregnancy outcomes. However, it must also be remembered that these antioxidant and other micronutrients can be obtained via a healthy diet thereby negating the need for supplementation. Future strategies focussing on providing nutritional guidance specifically to pregnant women will be pivotal in helping to ensure optimal health of both mother and baby.

## Figures and Tables

**Table 1:** Requirement of micronutrient intakes for selenium, copper, zinc, manganese, vitamin C, and vitamin E.

		Selenium (*μ*g/d)	Copper (*μ*g/d)	Zinc (mg/d)	Manganese (mg/d)	Vitamin C (mg/d)	Vitamin E (*μ*g/d)
RDA^1^	Female adult	55	900	8	1.8	75	15
	Pregnancy	60	1,000	11	2	85	15
	Upper limit	400	10,000	40	—	2,000	1,000
RNI^2^	Female adult	60	1,200	7	1.4	40	—
	Pregnancy	75	1,500	7	—	50	—
NR^3^	Female adult	30	1,350	1	—	—	—
	Pregnancy	—	1,150	2	—	—	—

RDA: recommended dietary allowance; RNI: reference nutrient intakes; NR: normative requirement estimate. Values taken from ^1^Institute of Medicine [77, 79], ^2^Department of Health [80], and ^3^WHO/FAO/IAEA [81].
